# Identification and Characterization of Unique Subgroups of Chronic Pain Individuals with Dispositional Personality Traits

**DOI:** 10.1155/2016/5187631

**Published:** 2016-04-04

**Authors:** S. Mehta, D. Rice, A. McIntyre, H. Getty, M. Speechley, K. Sequeira, A. P. Shapiro, P. Morley-Forster, R. W. Teasell

**Affiliations:** ^1^Western University, London, ON, Canada N6A 3K7; ^2^St. Joseph's Health Care London, London, ON, Canada N6A 4V2; ^3^Lawson Health Research Institute, London, ON, Canada N6C 0A7

## Abstract

*Objective.* The current study attempted to identify and characterize distinct CP subgroups based on their level of dispositional personality traits. The secondary objective was to compare the difference among the subgroups in mood, coping, and disability.* Methods.* Individuals with chronic pain were assessed for demographic, psychosocial, and personality measures. A two-step cluster analysis was conducted in order to identify distinct subgroups of patients based on their level of personality traits. Differences in clinical outcomes were compared using the multivariate analysis of variance based on cluster membership.* Results.* In 229 participants, three clusters were formed. No significant difference was seen among the clusters on patient demographic factors including age, sex, relationship status, duration of pain, and pain intensity. Those with high levels of dispositional personality traits had greater levels of mood impairment compared to the other two groups (*p* < 0.05). Significant difference in disability was seen between the subgroups.* Conclusions.* The study identified a high risk group of CP individuals whose level of personality traits significantly correlated with impaired mood and coping. Use of pharmacological treatment alone may not be successful in improving clinical outcomes among these individuals. Instead, a more comprehensive treatment involving psychological treatments may be important in managing the personality traits that interfere with recovery.

## 1. Introduction

Chronic pain is a significant problem for many individuals as it interferes with one's activities of daily living due to its impact on exercise, sleep, relationships with others, and independence [1]. Furthermore, it has a direct societal impact due to the high costs to the health care system; one report estimates the annual cost of prescription medications alone to be approximately $17.8 billion in the United States [2]. Underlying diseases such as fibromyalgia, multiple sclerosis, arthritis, migraine headaches, and cancer are all common causes of chronic pain [3]; however, musculoskeletal damage (e.g., low-back pain) is the major causative factor with the highest prevalence of chronic pain [3]. In a 2006 European survey, approximately 19% of the study population had experienced chronic pain [4]; this is similar to Western rates of chronic pain as well [5]. Chronic pain differs from acute pain in that the latter typically lasts days to weeks while the former often lasts months to years [6]. Many individuals with acute pain recover within a month of their pain onset, but for the remaining subset of individuals who experience chronic unrelenting pain, they may be acutely resistant to recovery despite medical intervention.

There exist a number of modalities to ameliorate pain, which primarily target the underlying cause. These include pharmacological (e.g., analgesics, anti-inflammatories, and anticonvulsants) and nonpharmacological (e.g., physical therapy and electrical stimulation) interventions and have varying levels of success [7]. However, it has been recognized that recovery depends not only on physical factors but on psychological factors as well [8]. Treatment for chronic pain is often multimodal, involving a variety of therapies including those that are psychologically based that aim to improve coping and self-efficacy, for example, Cognitive Behavioural Therapy [9]. Research has demonstrated a clear association between psychological factors such as depression and anxiety and the chronic pain recovery continuum [10, 11].

Additional research has shown other psychological factors, including personality traits, impact of the chronicity, and strength of chronic pain [12]. Trait theory suggests that one's dispositional personality is the composition of a large number of broad traits (i.e., behaviour, thoughts, and emotions) that are habitual in nature [13]. Therefore, the way in which individuals experience and recover from pain is influenced by their dispositional personality traits. Many clinicians have observed that hospital outpatients with chronic pain often demonstrate a pattern of thinking and coping suggestive of underlying obsessive personality traits. This style has variably been called “ergomania,” “high action-proneness,” “physical hyperactivity,” or “counterdependency” and has been framed as a predisposing factor or diathesis for poor coping and poor adjustment [14]. With regard to chronic pain, these traits may include dispositional traits such as those involved in obsessive personality including experiential avoidance (EA), anxiety sensitivity (AS), inability to relax, excessive worrying, and perfectionism [15].

A new realm of treatment for chronic pain suggests acceptance as a way of allowing “individuals to move toward their goals or act on their values while contacting pain, difficult thoughts, feelings, and memories, without defense” (p. 145) [16]. Experiential avoidance, the opposite of acceptance, is the process by which individuals avoid participating in activities, thoughts, and feelings surrounding the pain. Individuals with this trait-like characteristic have been shown to have lower thresholds of pain endurance and tolerance, as well as slower recovery from pain events [17]. Anxiety sensitivity, excessive worrying, and an inability to relax are all traits that contain an element of fear. Anxiety sensitivity is the fear of anxiety-related sensations (i.e., somatic, cognitive, or social) [18]. In a study assessing 125 individuals attending a pain clinic, measures of anxiety sensitivity were associated with greater levels of pain, disability, and distress [18]. Few studies have examined individual dispositional personality traits as they relate to chronic pain beyond causal risk factors or noncausal markers; furthermore, their study in combination has yet to be conducted.

The importance of personality traits in relation to chronic pain is now recognized not only by researchers but by health care professionals as well. From an individual perspective, the ultimate goal is reduction in pain to improve one's quality of life; therefore, all factors that play a role in the recovery process including those that are physical or psychological in nature should be considered when developing a treatment plan. Examining dispositional personality traits may assist in identifying individuals in the acute or subacute stage who are at risk of potentially developing chronicity or long term disability and help target early intervention. Therefore, in a sample of people with chronic pain, the objectives of this study were to (1) conduct a cluster analysis to identify dispositional personality trait subgroups (i.e., EA, AS, inability to relax, excessive worrying, and perfectionism) and (2) determine whether these subgroups differed in measures of mood, coping, and pain intensity.

## 2. Methods

### 2.1. Participants and Procedure

Participants were recruited from an academic specialist pain outpatient clinic in London, Ontario. Participants had to meet the following inclusion criteria: be admitted to the outpatient pain clinic, have a diagnosis of chronic pain (≥3 months), and be between 18 and 65 years old. Given that this study involved the completion of self-report questionnaires, patients with an inability to read and write in English were excluded. Patients were not compensated for their participation in this study.

Patients who met the inclusion criteria were mailed a questionnaire booklet two weeks prior to their scheduled appointment at the pain clinic. Subsequently, a research assistant contacted the patients by telephone to answer the participants' questions and ensure that if patients were interested in participating, they signed the consent form prior to completing questionnaires. The research assistant then instructed the patients who signed the consent forms to complete the booklet prior to their appointment. The booklet consisted of the following questionnaires: Anxiety Sensitivity Index (ASI), Acceptance to Action Questionnaire (AAQ), average pain intensity, Depression Anxiety Stress Scales-Short Form (DASS-SF), Frost Multidimensional Perfectionism Scale (FMPS), Pain Catastrophizing Scale (PCS), Penn State Worry Questionnaire (PSWQ), Reactions to Relaxation and Arousal Questionnaire (RRAQ), and Pain Disability Index (PDI). Participants were asked to arrive to their scheduled clinic appointment half an hour before their appointment was scheduled to begin. Upon arrival at the clinic, the research assistant collected the questionnaire booklet and made an attempt to complete and clarify all answers left blank by the participant. Background information related to the patient's demographic factors was also collected. All procedures were approved by the University of Western Ontario Health Sciences Review Board.

### 2.2. Cluster Variable Measures

#### 2.2.1. Anxiety Sensitivity Index (ASI)

The ASI [19] is a 16-item measure of the fear of anxiety-related symptoms composed of three factors: fear of the somatic symptoms of anxiety, fear of mental incapacitation (“cognitive dyscontrol”), and fear of negative social repercussions of anxiety [20]. Each item is rated on a five-point Likert scale ranging from 0 (very little) to 4 (very much). The instrument's psychometric properties and predictive validity have been well established [19].

#### 2.2.2. Acceptance and Action Questionnaire (AAQ)

The AAQ [21] is a 9-item self-report measure of experiential avoidance or the unwillingness to remain in contact with distressing private experiences (body sensations, emotions, and thoughts) and the inclination to alter the form or frequency of these experiences. It yields a single factor solution and is correlated with a wide range of negative behavioural and physical health outcomes [21]. The AAQ demonstrates adequate validity and reliability scores [21].

#### 2.2.3. Frost Multidimensional Perfectionism Scale (FMPS)

The FMPS [22] contains subscales measuring six different dimensions of perfectionism. In the present study, we used the total score with the parental standards and criticism subscales omitted. Research suggests that the concerns about mistakes and doubts about actions subscales are related to negative affectivity and reflect “maladaptive” perfectionism, while the high standards and need for organization subscales are unrelated or inversely related to negative affectivity [23–25].

#### 2.2.4. Penn State Worry Questionnaire (PSWQ)

The PSWQ is a 16-item measure of the frequency and intensity of worry that yields a single score [26]. The PSWQ is considered a trait-like feature where patients report items on a scale from 1 (“not at all typical of me”) to 5 (“very typical of me”). This measure yields a single factor structure and has good predictive validity [27].

#### 2.2.5. Reactions to Relaxation and Arousal Questionnaire (RRAQ)

The RRAQ is a nine-item factor analysis derived measure of fear of relaxation [28]. Participants rate the applicableness and accuracy of each item from 1 (“not at all”) to 5 (“very much so”). This measure has high retest reliability and strong convergent and discriminant validity [29].

### 2.3. Outcome Measures

#### 2.3.1. Average Pain Intensity

Pain ratings for current, least, average, and worst pain were summed to yield an aggregate pain intensity score. The scale ranged from 0 to 10 with 0 indicating no pain and 10 indicating intense pain. This composite pain intensity score has been shown to be a very reliable measure of pain intensity in chronic pain patients and has been used in recent research [30].

#### 2.3.2. Depression Anxiety Stress Scales-Short Form (DASS-SF)

The DASS-SF [31] is a 21-item self-report that measures feelings of depression, anxiety, and stress over the previous week. This short form scale is an abbreviated version of the 42-item scale developed by P. F. Lovibond and S. H. Lovibond [31] and has the same good to excellent psychometric properties as the original scale [32].

#### 2.3.3. Pain Catastrophizing Scale (PCS)

The PCS contains 13 items assessing the tendency to misinterpret and exaggerate the threat value of pain sensations. It has good psychometric properties and includes 3 main factors: rumination, magnification, and helplessness [33].

#### 2.3.4. Pain Disability Index (PDI)

The PDI [34] is a 7-item questionnaire asking participants to rate the degree to which pain interferes with functioning across seven domains: family/home responsibilities, recreation, social activity, occupation, self-care, life support activity, and sexual functioning. Scales are summed to derive a total disability index. In this study, the sexual functioning item was omitted.

### 2.4. Data Analysis

Whereas factor analysis uses intercorrelations among variables to form a smaller number of factors, cluster analysis is used to assign people to groups, or “clusters,” that share certain similarities. A two-step cluster analysis was performed using SPSS to organize observations into two or more mutually exclusive groups, where members of the groups shared properties in common. Five clustering variables were used in the analysis: ASI, AAQ, FMPS, RRAQ, and PSWQ. The log-likelihood distance measure was used, with subjects assigned to the cluster leading to the largest likelihood. The number of clusters was not predetermined. The Bayesian information criterion was used to judge adequacy of the final solution. Differences in sample demographic characteristics (age, gender, relationship status, duration of pain, and average pain intensity) were compared according to cluster membership using multivariate analysis of variance for continuous variables and *χ* ^2^ tests for categorical variables in order to characterize the resulting clusters. Prior to performing multivariate analyses, preliminary tests were conducted to confirm that there were no violations of assumptions. Specifically, before conducting the MANOVA we tested for normal distribution, linearity, homogeneity of variance and covariance, outliers, and multicollinearity. A multivariate analysis of variance (MANOVA) was conducted on continuous outcome measures (DASS-SF total, DASS-SF depression, DASS-SF anxiety, DASS-SF stress, and pain disability) according to cluster membership. Pairwise comparisons were conducted with Bonferroni adjustment. SPSS version 23.0 (Chicago, IL) was used for all tests performed, with the significance level set at alpha 0.05, and all tests were two-tailed.

## 3. Results

A total of 383 patients were eligible during the study period, of whom 229 (59.79%) agreed to participate. The sample was predominantly female (64.2%) and had an average age of 45.6 years (SD = 11.5). The majority of individuals were married or in a serious relationship (73.1%) and had experienced chronic pain for an average of 6.39 years (SD = 6.59; Table 1). Patients self-reported a number of sources of pain which greatly varied between patients. Bulged disc (14%), back pain (12%), and fibromyalgia (11%) were the three most commonly reported primary sources of pain among patients. Whiplash (6%) and nerve pain (6%) were also reported among a number of patients.

**Figure 1:**
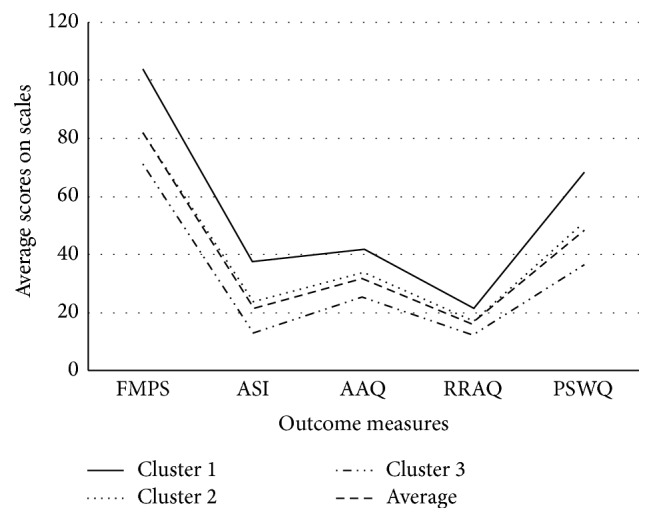
Distribution of dispositional traits among the 3 clusters. Note: AAQ = Action and Acceptance Questionnaire, ASI = Anxiety Sensitivity Inventory, FMPS = Frost Multidimensional Perfectionism Scale, PSWQ = Penn State Worry Questionnaire, and RRAQ = Reactions to Relaxation and Arousal Questionnaire.

The two-step cluster analysis resulted in 3 clusters, without exclusion of cases. Inspection of the Bayesian information criterion confirmed the three-cluster solution (change in the Schwarz's Bayesian criterion = −29.84; distance measures ratio = 2.1). In order to validate the clusters, a one way MANOVA was conducted. The mean scores of the FMPS, ASI, AAQ, RRAQ, and PSWQ were significantly different among the three clusters (*p* < 0.001) with Cluster 1 (*n* = 37) scoring significantly higher (≥1 SD above mean) on each measure than Cluster 2 (*n* = 104) and Cluster 3 (*n* = 88; Figure 1). Cluster 1 scored the highest on each measure, while Cluster 3 scored the lowest on each measure (≤1 SD above mean) and was significantly lower than Clusters 1 and 2 (*p* < 0.05) on all measures. Demographic (age, gender, relationship status, duration of pain, and average pain intensity) variables were comparable among all clusters (*p* > 0.05).

As seen in Table 2, there was a significant main effect of clusters on DASS-SF total (*F*(2,225) = 25.6, *p* < 0.001), DASS-SF depression (*F*(2,225) = 21.7, *p* < 0.001), DASS-SF anxiety (*F*(2,225) = 24.1, *p* < 0.001), DASS-SF stress (*F*(2,225) = 24.6, *p* < 0.001), PCS (*F*(2,225) = 25.3, *p* < 0.001), and PDI (*F*(2,223) = 3.1, *p* < 0.03). As seen in Table 3,* post hoc* analysis with Bonferroni adjustment resulted in a number of significant differences in mood (DASS-SF), pain catastrophizing (PCS), and pain disability (PDI) among clusters. Cluster 1 scored significantly higher than Clusters 2 and 3 on total DASS-SF score, while Cluster 2 scored significantly higher than Cluster 3, suggesting that Cluster 3 experienced the least distress. This finding was also supported by the subscale scores, whereby Cluster 1 scored significantly higher than Clusters 2 and 3 on DASS-SF anxiety, depression, and stress scales, while Cluster 2 also scored significantly higher on each subscale in comparison to Cluster 3. This pattern of scores between clusters was similar for the PCS and PDI scales; however, Cluster 1 did not significantly differ from Cluster 2 for any of these scales (see Table 3).

## 4. Discussion

The current study demonstrated that, in a group of individuals with CP, subgroups can be identified based on specific dispositional variables including perfectionism, anxiety sensitivity, experiential avoidance, inability to relax, and maladaptive worrying. The results from this study add to the existing data on CP and further the clinical impression of CP as a heterogeneous disorder. A cluster analysis identified 3 meaningful subgroups of CP individuals. Despite there being no difference between the subgroups in terms of demographic factors such as age, duration of pain, and average pain intensity, the 3 groups differed over the time course in clinical outcomes. Almost half the sample consisted of individuals with moderate levels of dispositional traits, pain catastrophizing, and pain disability whose level of mood impairment was high (Cluster 2). These may represent a “typical” patient seen in a tertiary pain center setting. The next largest group (Cluster 3) was those with low levels of the dispositional traits, mood impairment, pain catastrophizing, and pain disability. This group may represent those who have learned to cope more effectively with CP. The smallest group (Cluster 1) had very high levels of dispositional traits, mood impairment, pain catastrophizing, and pain disability. This group may represent those at highest risk for long term impairment among the CP population.

Inconsistent with early research on dispositional personality traits among the CP population [35, 36], the present study did not find a relationship between dispositional personality traits and pain intensity. The difference may be due to the use of general personality profile analysis by the earlier studies using Minnesota Multiphasic Personality Inventory (MMPI) or NEO-Five-Factor Inventory, while the current study uses specific personality traits. Use of specific personality measures allows for validated outcome tools to measure the trait of interest rather than using a generic inventory. This may allow for a more specific analysis of the traits of interest. However, our study produced similar results to those seen in a recent study which used the NEO-Five-Factor Inventory in a sample of patients with fibromyalgia [37]. In this study, two clusters emerged, with one cluster characterized by maladaptiveness. Patients in this cluster had a predisposition to experience affective distress and poorly manage social conflicts. These patients also scored significantly higher on neuroticism and lower on extraversion, openness to experience, agreeableness, and conscientiousness in comparison to the second cluster [37]. Multivariate analyses comparing the two clusters found that the maladaptive cluster had significantly higher scores for depression, anxiety, and each pain catastrophizing subscale. These significant differences between clusters depression, anxiety, and the pain catastrophizing rumination subscale were also present at six-month follow-up [37].

The study found that mood impairment was significantly higher among those with high levels of dispositional traits compared to those with moderate or low levels of dispositional traits. This is consistent with other studies which have found a strong link between personality traits and psychological distress among individuals with chronic pain [38, 39]. BenDabba et al. [40] reported that though personality traits and distress were highly correlated before treatment of CP, after treatment the relationship weakened. Furthermore, the study found that at 1- and 2-year follow-ups, the relationship between psychological distress and pain severity became stronger than that at baseline, while the relationship between distress and personality became weaker. This finding may indicate that treatment changes an individual's perception of pain factors, thereby changing psychological distress, while personality stays constant. Hence, it may be possible to help improve mood related outcomes even among those with high levels of dispositional traits.

Pain catastrophizing was significantly higher among the high and moderate groups compared to the low groups. However, no significant difference in catastrophizing was seen between the high and moderate groups. It may be that only those with low levels of the dispositional traits examined are able to cope with the pain experience without having exaggerated negative interpretations. Catastrophizing behaviour has been shown to be negatively correlated with an accepting coping style [41–44]. It may be that those individuals in the high and moderate groups of personality traits had prolonged rumination and were unable to accept their pain due to their higher levels of perfectionism and excessive worrying; thus significantly impacting other secondary pain outcomes. Hayes et al. [45] found that decreasing number of negative thoughts about pain and increasing pain acceptance were related to improvement in quality of life outcomes.

Similar to catastrophizing, pain disability was significantly lower only among those in the lowest group of dispositional personality traits compared to the other two groups, while no significant difference was seen between the high and moderate groups. Ramírez-Maestre and Esteve [46] also found that personality traits including neuroticism, anxiety sensitivity, and experiential avoidance were risk factors for increased probability of disability among individuals with chronic pain. Mehta et al. [47] identified subgroups of chronic pain individuals based on their levels of anxiety sensitivity and experiential avoidance. The study found that individuals with high levels of both factors had high levels of disability. Though perfectionism and excessive worrying were important factors in catastrophizing behaviour, anxiety sensitivity and experiential avoidance may play an integral role in disability related outcomes.

The current study has several limitations. First, unlike previous studies that used personality inventories such as the MMPI to identify clusters, we used specific traits. This limited our ability to examine all traits that may influence pain related outcomes. Secondly, this study may have a referral bias. Patients were recruited through convenience sampling of tertiary pain clinics. These individuals may already be at risk for greater psychological distress and disability. Hence, these results may not be generalizable to those individuals with chronic pain in the community. Further, cluster analyses require the authors' interpretation of formed groups which can be subjective. Lastly, due to the lack of a control group, causal relationships are not discernable through this study.

Despite these limitations, the current study has several implications. First, the study demonstrates the existence of a high risk subgroup of CP individuals that may be difficult to manage with pharmacotherapy alone due to the increased level of psychomorbidity and issues with personality traits. The information gained from these descriptive clusters may also help to inform health care providers on how to intervene with patients that may need help reshaping their illness perceptions. Use of acceptance based strategies among those in the high and moderate group may help reduce their long-term negative outcomes. Further studies to replicate these findings are necessary to determine whether the clusters from this study are generalizable to other CP populations.

## Figures and Tables

**Table 1:** Demographic and clinical characteristics of study population and participants subdivided into the three cluster subgroups.

	Study population	Cluster 1	Cluster 2	Cluster 3	*p* (among clusters)
*N*	229	37	102	83	
Mean age (SD)	45.4 (11.5)	42.8 (10.5)	46.8 (12.4)	44.9 (10.6)	>0.05
Sex (M%)	35.8	18.9	37.8	41.0	>0.05
Relationship status (%)					
Single	12.8	11.4	12.2	14.0	>0.05
Married or in a serious relationship	73.1	62.9	74.5	75.6	
Divorced, separated, or widowed	14.2	25.7	13.3	10.5	
Years of significant pain (SD)	6.4 (6.6)	6.8 (4.9)	7.6 (8.9)	4.8 (3.2)	>0.05
Average pain intensity	6.4 (1.9)	6.5 (1.6)	6.4 (1.8)	6.3 (1.8)	>0.05
FMPS (SD)	81.1 (17.4)	103.5 (13.6)	81.7 (14.7)	71.0 (11.9)	<0.001
ASI (SD)	22.0 (12.4)	37.4 (12.9)	23.9 (9.2)	13.2 (7.2)	<0.001
AAQ (SD)	32.1 (7.8)	41.7 (5.3)	34.3 (4.7)	25.6 (6.1)	<0.001
RRAQ (SD)	16.1 (5.6)	21.7 (4.9)	17.3 (4.6)	12.4 (4.2)	<0.001
PSWQ (SD)	13.6 (48.4)	68.2 (7.7)	51.3 (8.0)	36.8 (8.4)	<0.001

**Table 2:** Mean values (standard deviation) in mood, catastrophizing, and pain disability among the cluster subgroups.

	Cluster 1	Cluster 2	Cluster 3	*F*	*p*
DASS total	33.1 (14.7)	26.5 (11.9)	17.3 (11.0)	25.6	**<0.001**
DASS depression	10.9 (5.6)	8.7 (4.8)	5.3 (4.0)	21.7	**<0.001**
DASS anxiety	11.5 (4.9)	9.2 (4.2)	6.2 (4.0)	24.1	**<0.001**
DASS stress	11.4 (5.5)	8.8 (4.2)	5.8 (4.0)	24.6	**<0.001**
PCS	42.7 (9.7)	37.9 (10.1)	28.8 (9.2)	25.3	**<0.001**
PDI	38.6 (9.0)	36.4 (10.5)	34.1 (12.6)	3.1	**<0.03**

Bold values denote *p* < 0.05. DASS = Depression Anxiety Stress Scale; PCS = Pain Catastrophizing Scale; and PDI = Pain Disability Index.

**Table 3:** *Post hoc* analysis mood, pain catastrophizing, and pain disability among clusters subgroups.

	Cluster #	Cluster 2		Cluster 3	
		Mean difference (S.E)	*p*	Mean difference (S.E)	*p*
DASS Total	1	6.8 (1.9)	**0.002**	13.3 (1.9)	**0.002**
	2			6.8 (1.4)	**0.001**
Depression	1	2.4 (0.7)	**0.004**	4.7 (0.7)	**0.001**
	2			2.3 (0.5)	**0.001**
Anxiety	1	2.3 (0.7)	**0.003**	4.6 (0.7)	**0.001**
	2			2.3 (0.5)	**0.001**
Stress	1	2.4 (0.7)	**0.001**	4.6 (0.7)	**0.001**
	2			2.1 (0.5)	**0.001**
PCS total	1	2.7 (1.6)	0.216	9.7 (1.6)	**0.001**
	2			7.0 (1.2)	**0.001**
Helplessness	1	1.0 (0.8)	0.363	4.3 (0.8)	**0.001**
	2			3.3 (0.6)	**0.001**
Magnification	1	0.7 (0.4)	0.174	2.4 (0.4)	**0.001**
	2			1.7 (0.3)	**0.001**
Rumination	1	0.9 (0.6)	0.228	3.1 (0.6)	**0.001**
	2			2.2 (0.4)	**0.001**
PDI	1	0.63 (1.9)	0.942	3.7 (1.0)	**0.042**
	2			3.1 (0.8)	**0.034**

*Note*. Bold values denote *p* < 0.05, S.E = standard error; DASS = Depression Anxiety Stress Scale; PCS = Pain Catastrophizing Scale; and PDI = Pain Disability Index.
